# A case report of *Pasteurella multocida* meningitis in a patient with non-traumatic skull base defect

**DOI:** 10.1016/j.idcr.2020.e00991

**Published:** 2020-10-23

**Authors:** Mohamed Kamal Sabra, Adeel Ahmad Khan, Musaed Al Samawi, Yasser El Deeb

**Affiliations:** a Department of Internal Medicine, Al Khor Hospital, Hamad Medical Corporation, Qatar; b Department of Infectious Diseases, Al Khor Hospital, Hamad Medical Corporation, Qatar

**Keywords:** Meningitis, *Pasteurella multocida*, CSF rhinorrhea, Skull defect

## Abstract

**Background:**

*Pasteurella multocida* is a gram-negative coccobacillus that is primarily found in oropharynx of dogs, cats and other animals. It causes infections in human beings through contact with animal saliva in the form of licks, bites and scratches of animals colonized by the bacteria. Meningitis due to *Pasteurella multocida* is rare in immunocompetent individuals. We report a case of meningitis due to *Pasteurella multocida* in an immunocompetent patient.

**Case report:**

A 30-year-old gentleman presented with 2-day history of fever and neck stiffness. 6 weeks earlier, he was treated as a case of bacterial meningitis. During that hospital stay, he was diagnosed to have bony defect in the sellar floor based on MRI head performed to evaluate for a prolonged history of CSF rhinorrhea. He was discharged and scheduled for an elective endoscopic endonasal/open repair of the skull base defect after resolution of meningitis. CSF findings during current admission also showed features of bacterial meningitis. CSF culture showed *Pasteurella multocida* sensitive to penicillin, ampicillin and ceftriaxone. Retrospective history revealed patient’s contact with stray cats as he used to feed them but there was no history of licks, bites. He was treated with intravenous ceftriaxone 2 g twice a day for 14 days with complete resolution of his symptoms.

**Conclusion:**

*Pasteurella multocida* is an important cause of bacterial meningitis in patients with skull defect. Patients with traumatic or non-traumatic bony defect of skull should avoid contact with dogs and cats to prevent the spread of infection the central nervous system.

## Introduction

*Pasteurella multocida* is a gram-negative coccobacillus that is primarily found in oropharynx of dogs, cats and other animals. It is known to cause infections in human beings through contact with animal saliva most commonly in the form of licks, bites and scratches of animals colonized by the bacteria [1,2]. Diseases caused by *Pasteurella* in human beings include localized soft tissue infection, osteomyelitis, septic arthritis, pneumonia, lung abscess, bacteremia, endocarditis and meningitis [2]. We report a case of meningitis caused by *Pasteurella multocida* in an immunocompetent patient who had bony defect in the sellar floor acting as a risk factor for spread of organism causing meningitis.

## Case report

A 30 years old male patient presented to the hospital with a 2-day history of high-grade fever and headache. Headache was diffuse, moderate to severe in intensity, associated with pain and stiffness in neck, photophobia and vomiting. Condition was not associated with blurring of vision, double vision, vomiting, loss of consciousness or convulsions. There was no history of sore throat, cough, ear pain, ear discharge, motor weakness, sensory weakness, hearing difficulty, altered level of consciousness, abnormal jerky movements or skin rashes. Patient also reported persistent colorless discharge from nose and postnasal drip for 4 months. There was no history of ear fullness, persistent headache or head trauma. Patient was non-smoker and there was no history of alcohol use.

**Image 1:**
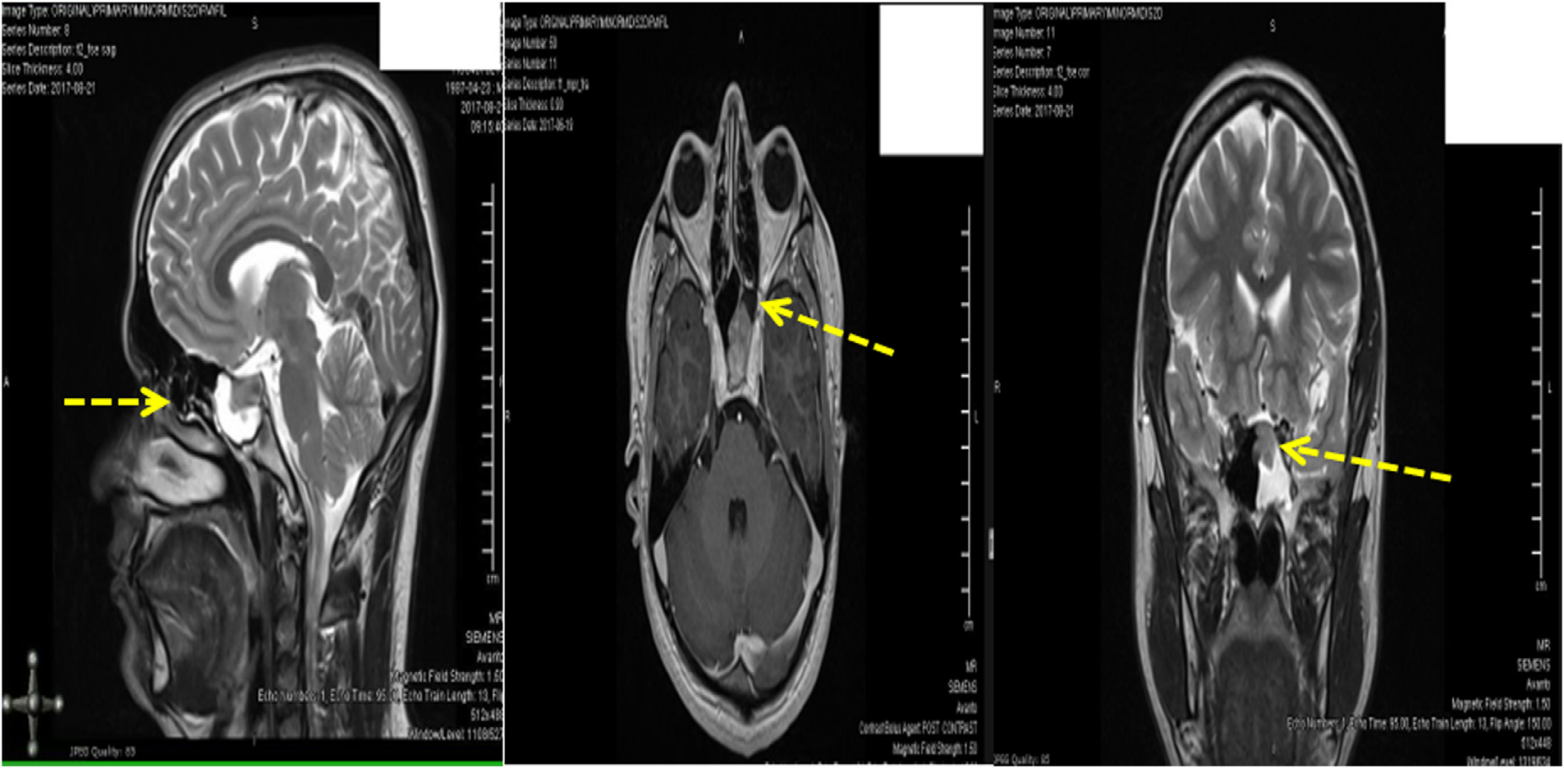
MRI HEAD showing large bony defect in the sellar floor with soft tissue in the anterior aspect of the sella with infrasellar extension into the left sphenoid sinus. (Dashed yellow arrow).

6 weeks ago, patient was discharged from hospital after being treated as bacterial meningitis. The CSF examination for viral PCR and bacterial gram stain/culture was negative. He was treated with a 14-day course of intravenous (IV) ceftriaxone. He had complete recovery of symptoms without any neurological sequalae. During hospital stay at that time, he underwent MRI head which showed large bony defect in the sellar floor with soft tissue in the anterior aspect of the sella with infrasellar extension into the left sphenoid sinus Image 1). Neurosurgery and otorhinolaryngology teams were consulted, and an elective endoscopic endonasal/open repair of the skull base defect was planned as outpatient after resolution of meningitis. Patient still had persistent rhinorrhea during this presentation as he did not undergo repair of bony defect of sellar floor.

Physical examination revealed a young adult well oriented to time, place and person. He was febrile with temperature of 38.9 degrees Celsius. Pulse, blood pressure and respiratory rate was normal. There were no skin rashes or scratch marks. GCS was 15/15. He had normal cranial nerves, motor and sensory examination. Neck stiffness, Kernig’s and Brudzinski’s signs were positive. Cardiovascular, chest and abdominal examination was normal.

**Image 2:**
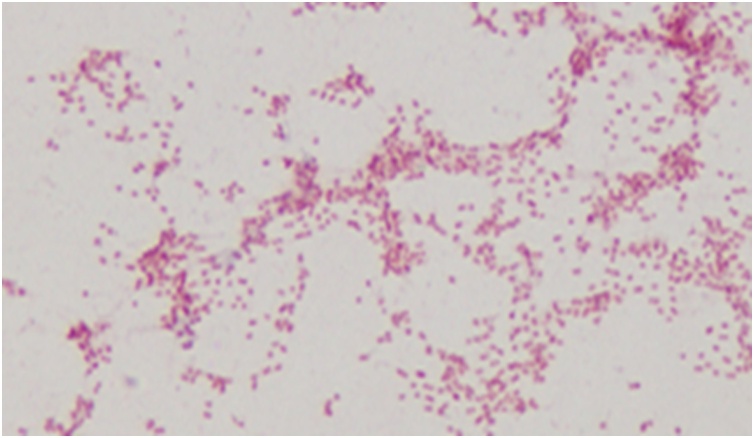
Gram Stain of CSF showed Gram negative coccobacilli (*Pasteurella multocida*).

Complete blood count revealed neutrophilic leukocytosis with raised C reactive protein. Urea, creatinine, electrolytes and liver function tests were normal. Lumbar puncture was performed and cerebrospinal fluid (CSF) examination showed high white blood cell count with predominance of neutrophils, high protein and low glucose (Table 1). Viral PCR of CSF was negative. CSF culture showed gram negative coccobacilli (Image 2) identified as *Pasteurella multocida* sensitive to ceftriaxone, ampicillin and penicillin. Blood cultures were negative. After confirmation of the organism, a retrospective history of contact with dogs, cats or other animals was taken which revealed that he often used to feed stray cats who lived around his house. However, there was no history of cat scratches, bites, licks or kisses on any part of the body. Patient was treated with IV ceftriaxone 2 g twice a day for 14 days with complete resolution of his symptoms. He was offered surgical correction of his skull defect, but he preferred to do it in his home country. He was discharged with advice to avoid contact with dogs and cats till surgical correction of bony defect of skull.

**Table 1:** Cerebrospinal fluid Examination.

	Result	Reference
White blood cells	960/μl with 88 % neutrophils and 8 %lymphocytes	<5cells/μl
Protein	1.36 gm/L	Protein <0.45grm/L
Glucose	2.2 mmol/L	CSF/serum ratio >0.6
	(Serum glucose 5.7 mmol/L	
	CSF glucose/serum glucose ratio = 0.38)	
AFB smear and TB PCR	Negative	
Meningitis viral panel (PCR)	Negative	
Culture of CSF	Gram Negative coccobacilli	
	(Image 2) confirmed by MALDI-TOF to be *Pasteurella multocida*	

AFB: Acid Fast Bacillus.

PCR: Polymerase Chain Reaction.

TB: Tuberculosis.

CSF: Cerebrospinal Fluid.

MALDI-TOF: Matrix-assisted laser desorption ionization-time-of-flight.

## Discussion

Five species of *Pasteurella* genus have been identified as a cause of infections in humans. These include *P. multocida*, *P. septica*, *P. canis*, *P. stomatis*, and *P. dagmatis*. Out of these, *P. multocida* is the commonest to cause infection in human beings [3]. Pasteurella meningitis is most commonly seen in extremes of ages, less than 1 years and more than 55 years of age [4,5]. Patients with conditions that lead to compromise in immune status, for example liver cirrhosis, patients on dialysis and HIV infection, have an increased risk of developing complications of Pasteurella infection [[6], [7], [8]].

Meningitis caused by *Pasteurella* is rare in immunocompetent patients. A history of animal contact can be elicited in most cases. *Pasteurella multocida* meningitis can result after penetrating animal bite of the skull causing direct inoculation of the organism, bacteremia or by spread of infection from other sites in patients with skull fracture or who have gone neurosurgical intervention. Other methods of acquiring *Pasteurella multocida* infection include contact with oropharyngeal secretions by direct kissing or licking by the animal [9].

Cases of *Pasteurella multocida* meningitis in otherwise immunocompetent individuals have been reported in literature after neurosurgical procedures and trauma. Lee et al. described a case of ventriculoperitoneal shunt infection with *Pasteurella* [10]. Roberts et all also described a case of *Pasteurella multocida* meningitis in a patient with post traumatic basilar skull fracture [11]. Our patient had a history of non-traumatic CSF rhinorrhea due to bony defect in the sellar floor which, in the presence of positive history of animal contact, acted as a major risk factor for developing meningitis.

*P. multocida* is usually sensitive to penicillin, ampicillin and third generation cephalosporins [12]. IV Ampicillin 2 g every 4 hourly or IV ceftriaxone 2 g every 12 hourly are suitable initial options [13]. Optimum duration of treatment is from 14 days to 21 days. However, strains producing beta-lactamase have been identified as well and antibiotics should be tailored once susceptibility results are available [14].

Literature has reported attempts to establish link between the organism identified from the infected human and culprit animal [5,15]. In our case, our patient had contact with a stray cat and hence, it was not possible to identify the animal responsible for the transmission of infection.

## Conclusion

Our case emphasizes the importance of taking appropriate history of animal contact in cases of meningitis, which can point towards the identification of the culprit organism. *Pasteurella multocida* is an important cause of bacterial meningitis in patients with skull defect. Patients with traumatic or non-traumatic bony defect of skull should avoid contact with dogs and cats to prevent the spread of infection to the central nervous system till definitive surgical correction is performed. Furthermore, in immunocompetent patients with *Pasteurella* meningitis without any history of trauma or animal bite, an attempt should be made to identify an underlying bony defect that might be the predisposing factor for the spread of organism to the nervous system.

## Funding acquired

Open access funding for this article was provided by the Qatar National Library.

## Authors’ contribution

First and second author contributed equally to the manuscript. MKS was involved in management of case. He wrote the initial manuscript and conducted literature review. AAK wrote discussion and conducted literature review. He also critically reviewed the manuscript before submission. MAS and YED were involved in patient management and critically analyzed the manuscript.

## Declaration of Competing Interest

The authors report no declarations of interest.
